# Mediastinal lymph node enlargement in idiopathic pulmonary fibrosis: relationships with disease progression and pulmonary function trends

**DOI:** 10.1186/s12890-020-01289-2

**Published:** 2020-09-21

**Authors:** Giacomo Sgalla, Anna Rita Larici, Nicoletta Golfi, Mariarosaria Calvello, Alessandra Farchione, Annemilia Del Ciello, Francesco Varone, Bruno Iovene, Riccardo Manfredi, Luca Richeldi

**Affiliations:** 1 Dipartimento Scienze Gastroenterologiche, Endocrino-Metaboliche e Nefro-Urologiche, Unità Operativa Complessa di Pneumologia, Fondazione Policlinico Universitario “A. Gemelli” IRCCS, Rome, Italy; 2 Dipartimento Diagnostica per Immagini, Radioterapia Oncologica ed Ematologia, Fondazione Policlinico Universitario A. Gemelli IRCCS, Rome, Italy; 3 Dipartimento Universitario Scienze Radiologiche ed Ematologiche, Sezione di Radiologia, Università Cattolica del Sacro Cuore, Rome, Italy; 4 Università Cattolica del Sacro Cuore, Rome, Italy

**Keywords:** Idiopathic pulmonary fibrosis, Interstitial lung disease, Lymphadenopathy

## Abstract

**Background and objectives:**

Evidence of mediastinal Lymph Node Enlargement (LNE) on CT scan is a common finding in idiopathic pulmonary fibrosis (IPF). We sought to investigate whether the involvement of mediastinal lymph nodes is associated with accelerated disease progression, and explored the changes occurring in mediastinal lymph nodes during the radiological follow up of these patients.

**Methods:**

This retrospective study included IPF patients referred to a single ILD centre in Italy. A consensus-based assessment of mediastinal LNE on chest CT scan was performed by two thoracic radiologists. Kaplan-Meier curves and multivariate Cox proportional hazards regression were used to assess hazard ratios for mortality and disease progression (defined as categorical FVC decline ≥10%). The annualized rates of change in functional parameters for each patient were calculated using mixed linear models.

**Results:**

The study population consisted of 152 IPF patients, of whom 135 (89%) received antifibrotic treatment for IPF during the study follow up. Patients having evidence of 3 or more enlarged mediastinal lymph nodes on baseline CT scan showed increased rates of mortality (HR 5.03, 95% CI 1.86–13.62, *p* ≤ 0.001) and significant disease progression (HR 2.99, 95% CI 1.22–7.33, *p* = 0.17) as compared to patients without LNE, after adjusting for GAP stage. Among 62 patients with LNE who underwent a follow up CT scan of the chest and received antifibrotic treatment, 57 (92%) maintained evidence mediastinal LNE over time.

**Conclusions:**

Diffuse mediastinal lymph node involvement predicts clinically meaningful functional deterioration in patients with IPF.

## Background

The prognosis of Idiopathic pulmonary fibrosis (IPF), the most fatal among idiopathic interstitial pneumonias [1, 2], is generally poor with an estimated survival between 3 and 5 years from the time of diagnosis [3]. Despite two antifibrotic drugs, nintedanib and pirfenidone, proved to slow down the functional decline in IPF [4, 5], most patients still deteriorate despite treatment. As such, accurate monitoring of disease progression in IPF is crucial for predicting prognosis and optimizing management, including the appropriate timing of supportive care and the prompt referral for lung transplantation. Clinical findings, pulmonary function measures and computed tomography findings have been extensively studied to predict survival in IPF, either individually or by using composite scoring indices [6–9], however prognostication remains challenging in the individual patient due to the heterogeneity of IPF and the significant intra-patient variability of the disease behaviour [10]. Measurement of forced vital capacity (FVC) via spirometry has been accepted as the most feasible and reliable tool to monitor disease progression in these patients in clinical practice as well as in randomized controlled trials [11, 12], however it has not been proved of great utility in predicting future loss of pulmonary function [13, 14]. Visual evaluation and computer-based quantification of high-resolution computed tomography (HRCT) lung parenchymal patterns of fibrosis have been demonstrated to be strong predictors of outcome, particularly in IPF patients [7, 9, 15–17], however their use in clinical practice is still limited. The evidence of mediastinal lymphadenopathies on chest CT scans inpatients with interstitial lung disease (ILD) have drawn the attention of researchers over the years, due to the putative role played by inflammation and immunity processes in pulmonary fibrosis. The frequency of this finding has been reported in up to 70% of patients with a usual interstitial pneumonia pattern [18, 19], and in up to 58% of IPF patients [20, 21]. Although the biology underpinning mediastinal lymph node enlargement in these patients has never been clarified, the presence of LNE on chest CT scans has been associated with increased all-cause mortality in IPF and other ILD [22, 23], suggesting a role as useful prognostic factor in fibrotic lung disease. An ongoing randomized clinical trial is currently exploring the efficacy of a human monoclonal antibody directed against the B-cell activating factor (BAFF) receptor on B cells in IPF patients (clinicaltrials.gov identifier NCT03287414). The study recognizes the presence of hilar/mediastinal lymphadenopathies on HRCT as one of the key inclusion criteria, based on the hypothesis that lymph node involvement may identify IPF patients with pronounced immune activation and who are therefore more likely to respond to the investigational treatment.

To date however, it is unknown whether the enlargement of mediastinal lymph nodes may be used to identify patients with more rapid functional deterioration, or to indicate response to currently available antifibrotic treatments.

In this study, we retrospectively evaluated the impact of LNE on mortality and disease progression in a cohort of IPF patients, and explored the changes occurring in mediastinal lymph nodes during the radiological follow up.

## Methods

### Study population and groups

All patients referred to the outpatient ILD clinics at Fondazione Policlinico Universitario “A. Gemelli” IRCCS in Rome between January 2014 and November 2018 were retrospectively screened for inclusion in the study. Patients were included if they received a multidisciplinary diagnosis of IPF as per diagnostic guidelines [1, 3] and if they had performed a chest CT scan within 1 year from diagnosis. Patients were excluded if they had either concurrent pulmonary infection at the time of the CT scan, acquired immunodeficiency syndrome (AIDS), unstable chronic heart disease or history of malignancy with less than 5 years of negative follow up. Subjects with no available Pulmonary Functional Tests (PFTs) were also excluded. The study was approved by the local ethics committee of Fondazione Policlinico Universitario “A. Gemelli” IRCCS in Rome.

The presence (LNE+) or absence (LNE-) of at least one mediastinal lymph node with short-axis diameter ≥ 10 mm on chest CT scan at baseline, as reviewed by thoracic radiologists, was used to define the groups for this study.

### Collection of study data

The medical records at the ILD outpatient clinic Fondazione Policlinico Universitario “A. Gemelli” IRCCS in Rome were used to obtain clinical data up to April 2019. Demographics and medical history including smoking status, supplemental oxygen therapy, antifibrotic treatment and comorbidities were collected together with baseline and follow up lung function data such as absolute and percent predicted values of FVC and diffusion capacity for carbon monoxide (DL_CO_) were obtained. The Gender/Age/Physiology (GAP) score, that integrates patient-specific variables (sex, age, FVC, DLCO) [8], was calculated for each patient. The follow up time was calculated for each patient as the time interval between the first and the last functional test performed at the referral centre.

### Chest CT evaluation

The CT exams evaluated for this study were performed at different centres, with some variability in reconstruction parameters used and contrast medium administration. Since most of CT studies were performed for the analysis of the lung parenchyma, CT scans without contrast medium administration were the majority and were preferred for the analysis when available. Images with a maximum slice thickness of 5 mm (range: 1–5 mm), smooth reconstruction algorithm and soft tissue window setting (level 50/width 350 HU) were used for lymph node assessment. Mediastinal lymph node stations based on the International Association for the Study of Lung Cancer nomenclature were systematically assessed for enlarged lymph nodes [24]. Mediastinal lymph node measurements were provided using electronic calipers. Lymph nodes with a short-axis diameter ≥ 10 mm were reported as enlarged, as previously described in papers focused on ILD [22, 23]. The number and maximum short-axis diameter for each station as well as location (upper zones, lower zones, or both) of LNE were recorded. When available, follow up CTs were also reviewed for the presence of LNE to evaluate the longitudinal behaviour of LNE. Radiologic patterns were also assessed according to the current ATS/ERS/ALAT/JRS guidelines for diagnosis of IPF (UIP, probable UIP, Indeterminate for UIP and alternative diagnosis) [1]. All the evaluations were performed via consensus between two thoracic radiologists with expertise in ILD, blinded to clinical information of patients included in the study.

### Statistical analysis

Assuming a survival rate of 70% in the group without mediastinal LNE over a follow up period of 2 years, a sample of 148 patients was calculated as required to provide the study with 90% power to detect a 2.26-fold increased mortality risk in the group with mediastinal LNE with a two-sided significance level of 5%. The effect size was chosen based on a previous study investigating the impact of mediastinal LNE on survival in IPF [22].

Means with standard deviations and frequencies or percentages were used as descriptives for continuous and categorical variables, respectively. T-test for continuous variables and the chi-square test for categorical variables were used to perform between-group comparisons of demographics and clinical data. Kaplan–Meier curves and the log-rank test were used to analyse the rates of mortality from any cause during the follow up and the rates of disease progression as defined by a categorical FVC decline ≥10% measured at the time of the last available pulmonary function assessment.

Crude and adjusted hazard ratios and their 95% confidence intervals (CIs) were calculated using Cox proportional hazard regression. Observed baseline and 12(±4) months FVC and percent predicted DLco values were entered in a mixed linear model with fixed effects for presence of mediastinal LNE, time, gender, age and random effects for patient-specific intercept to predict the annualized rate of change in the parameters for each patient. The effect of mediastinal LNE on FVC and DLco changes over time was determined on the basis of the time-by-group interaction term from the mixed model. *P*-values less than 0.05 were considered statistically significant. All analyses were performed using SPSS (version 24, IBM, USA).

## Results

### Characteristics of study population

**Fig. 1:**
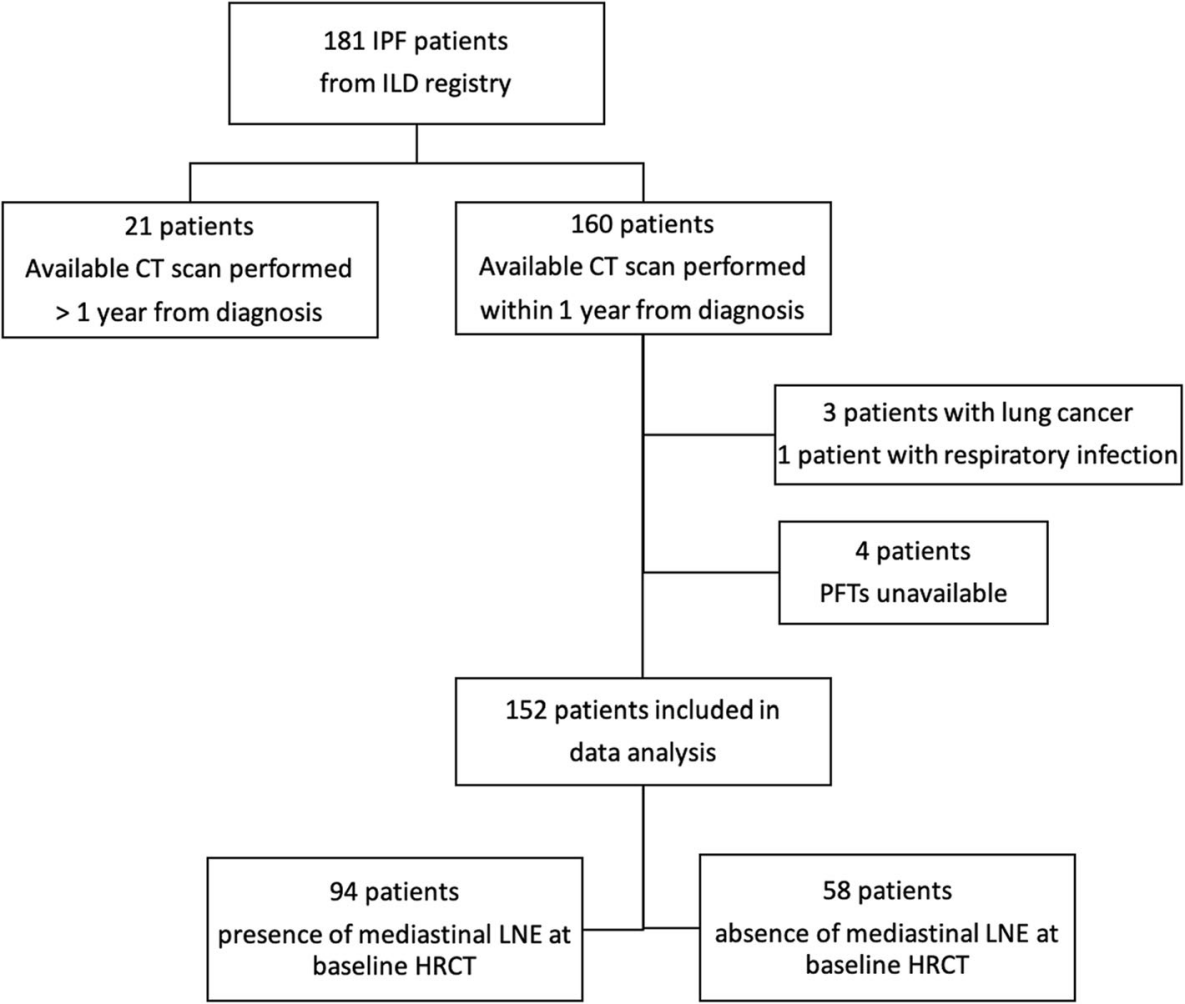
Flowchart of patient selection for the study

One hundred eighty-one IPF patients from the ILD clinic registry were screened for eligibility (Fig. 1). Overall, twenty-nine patients were excluded for unavailability of CT scan within 12 months from diagnosis (*n* = 21), history of thoracic malignancy (*n* = 3), respiratory infection at the time of diagnosis (*n* = 1) or for missing reports of baseline pulmonary function tests (*n* = 4). One hundred fifty-two IPF patients in the ILD registry formed the final study population. The average clinical follow up time was 19.08 months (SD 12.8). Ninety-four (62%) patients had at least one LNE on CT performed within a year from diagnosis, while 58 patients (38%) had no LNE present. Within patient with mediastinal LNE, 62 (66%) had two or more mediastinal lymph node with short-axis diameter ≥ 10 mm, while 31 (33%) had 3 or more LNE. In this group, the mean short-axis diameter of largest lymph node was 12.4 mm (SD 2.4). LNE was predominantly distributed in paratracheal stations (*n* = 93, 99% of LNE patients), while only 3 patients (3%) had LNE in the lower zones. The large majority of patients included in this study (*n* = 135, 89%) received antifibrotic treatment for IPF during the follow up.

Baseline characteristics of the study groups by presence of LNE on baseline CT scan are reported in Table 1. The two study groups did not present significant differences as to demographics. Patients without LNE had lower GAP stage (*p* = 0.038) and higher DLco % predicted at baseline (*p* = 0.023) as compared to LNE+ patients. Diabetes was a more frequent comorbidity among patients with LNE (25.8% vs 6.9%, *p* = 0.004).

**Table 1:** Baseline characteristics of patients without (LNE-) or with (LNE+) mediastinal lymph node enlargement on CT scan of the chest. Data are expressed as counts (%) or mean with standard deviation. Reported p-values were obtained via t-test for independent samples or Pearsons’ Chi-squared test as applicable. BMI = body mass index; FVC = forced vital capacity; DLco = diffusion lung capacity for carbon monoxide; 6MWD = 6-min walk distance; COPD = chronic obstructive pulmonary disease; OSAS = obstructive sleep apnea syndrome; GERD = gastroesophageal reflux disease; GAP = gender age physiology.

	N available observations	LNE- (*n* = 58)	LNE+ (*n* = 94)	*p* value
Age, years	152	76 (7.3)	74.7 (7.6)	0.277
Sex				0.627
Male	152	45 (77.6)	76 (80.9)	
Female		13(22.4)	18 (19.1)	
Smoking history				0.599
Current		0 (0)	1 (1.1)	
Former	148	34 (60.7)	60 (65.2)	
Never smoker		22 (39.3)	31(33.7)	
BMI	140	28.2 (3.9)	27.31 (4.5)	0.244
Use of oxygen therapy				0.405
No	137	51 (91.1)	70 (86.4)	
Yes		5 (8.9)	11(13.6)	
HRCT pattern				0.504
UIP	136	27 (52.9)	43 (50.6)	
Probable UIP		14 (27.5)	32 (37.6)	
Indeterminate for UIP		7 (13.7)	7 (8.2)	
Alternative diagnosis		3 (5.9)	3 (3.5)	
Anti-fibrotic treatment				0.064
No treatment	150	6 (10.5)	9 (9.7)	
Pirfenidone		30 (52.6)	32 (34.4)	
Nintedanib		21 (36.8)	52 (55.9)	
Follow up time (months)	140	21.2(13.4)	18.1 (12.4)	0.166
FVC volume, L	141	2.6 (0.7)	2.56 (0.73)	0.737
FVC % predicted	152	85.6 (21)	80.3 (19.5)	0.118
DLco % pred	142	58.9 (23.2)	50.1 (21.7)	0.023
SaO_2_% at rest	142	94.4 (2.2)	94.7 (2.7)	0.563
Comorbidities				
COPD	152	4 (6.9)	5 (5.3)	0.732
Emphysema	152	5 (8.6)	13 (13.8)	0.334
OSAS	152	6 (10.3)	8 (8.5)	0.704
Chronic heart disease	152	17 (29.3%)	32 (34)	0.544
Pulmonary hypertension	152	2 (3.4)	12 (12.8)	0.054
GERD	152	16 (27.6)	30 (31.9)	0.573
Anxiety/Depression	152	2 (3.4)	4 (4.3)	0.804
Diabetes	205	4 (6.9)	24 (25.8)	0.004
GAP Index				0.038
Stage I	148	25 (43.9)	35 (38.5)	
Stage II		30 (52.6)	40 (44.4)	
Stage III		2 (3.5)	16 (17.6)	
LNE number				
1			32 (34)	
2			31 (33)	
≥ 3			31 (33)	
Larger LNE (mm)	113	–	12.4 (2.4)	< 0.001

### All-cause mortality

**Fig. 2:**
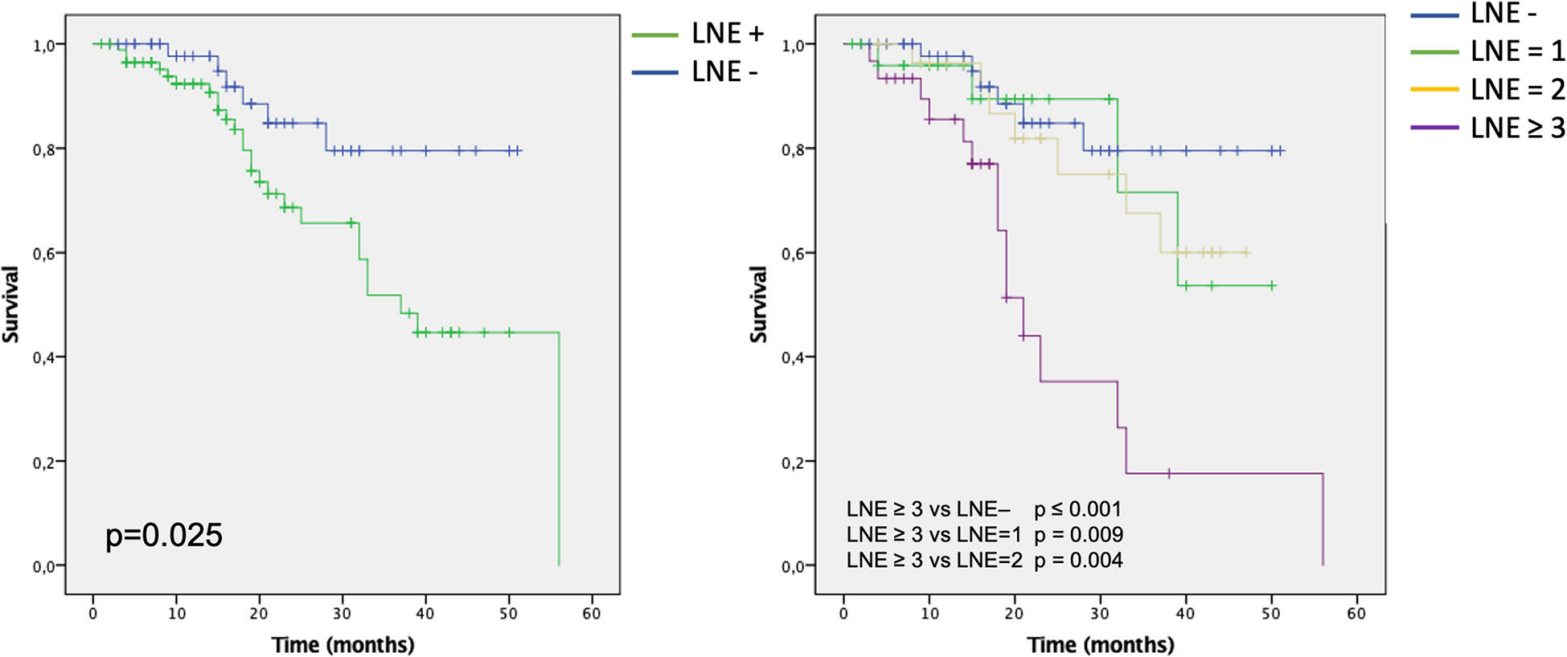
Kaplan-Meyer curves of mortality for patients with (LNE+) or without (LNE -) mediastinal lymph node enlargement (left) and for patients stratified according to the number of mediastinal enlarged lymph nodes (right). P values were obtained via log-rank test

IPF patients with evidence of mediastinal LNE on baseline CT scan of the chest had lower survival rates as compared to patients without LNE (median survival 37.8 months vs 44.5 months, log-rank *p* = 0.025) (Fig. 2). This corresponded to a 2.65-fold increased risk of mortality for LNE+ patients on Cox proportional hazard analysis (95% CI 1.09–6.46, *p* = 0.032) (Table 2). After stratification of the study population by number of enlarged mediastinal lymph nodes, the involvement of three or more lymph nodes was found significantly associated with worse survival as compared to each of the other groups (Fig. 2), with a 5.72-fold increased risk of mortality (HR 5.72, 95%CI 2.18–14.98, *p* < 0.001). Within patients with mediastinal LNE at baseline, increasing dimensions of the largest lymph node were also associated with higher mortality (HR 1.17, 95% CI 1.04–1.32, *p* = 0.01).

**Table 2:** – Cox proportional hazard regression analysis for all-cause mortality and disease progression. Disease progression was defined as death or absolute forced vital capacity (FVC) decline ≥10%. *Values of HR were adjusted for GAP (Gender, Age, Physiology) stage index. **within LNE+ patients

	Mortality				Disease progression			
	Unadjusted		Adjusted*		Unadjusted		Adjusted*	
	HR (95% CI)	*p* value	HR (95% CI)	p value	HR (95% CI)	p value	HR (95% CI)	p value
LNE + (vs LNE -)	2.65 (1.09–6.46)	0.032	2.39 (0.96–5.95)	0.062	1.75 (0.9–3.39)	0.097	1.87 (0.93–3.76)	0.078
Number of LNE (vs 0)								
1	1.49 (0.42–5.3)	0.538	1.1 (0.27–4.5)	0.894	1.43 (0.54–3.83)	0.472	1.65 (0.61–4.47)	0.326
2	1.62 (0.54–4.84)	0.386	1.55 (0.52–4.66)	0.436	1.41 (0.63–3.17)	0.405	1.53 (0.67–3.52)	0.313
≥ 3	5.72 (2.18–14.98)	< 0.001	5.03 (1.86–13.62)	0.001	2.67 (1.19–5.97)	0.017	2.99 (1.22–7.33)	0.017
Larger LNE**(mm)	1.17 (1.04–1.32)	0.01	1.14 (0.99–1.29)	0.058	1.05 (0.89–1.23)	0.57	0.93 (0.84–1.18)	0.928

When hazard ratios were adjusted for GAP stage [8], only the presence of 3 or more mediastinal enlarged lymph nodes maintained a strong association with poorer survival (HR 5.03, 95% CI 1.86–13.62, *p* ≤ 0.001) (Table 2).

### Pulmonary function

**Fig. 3:**
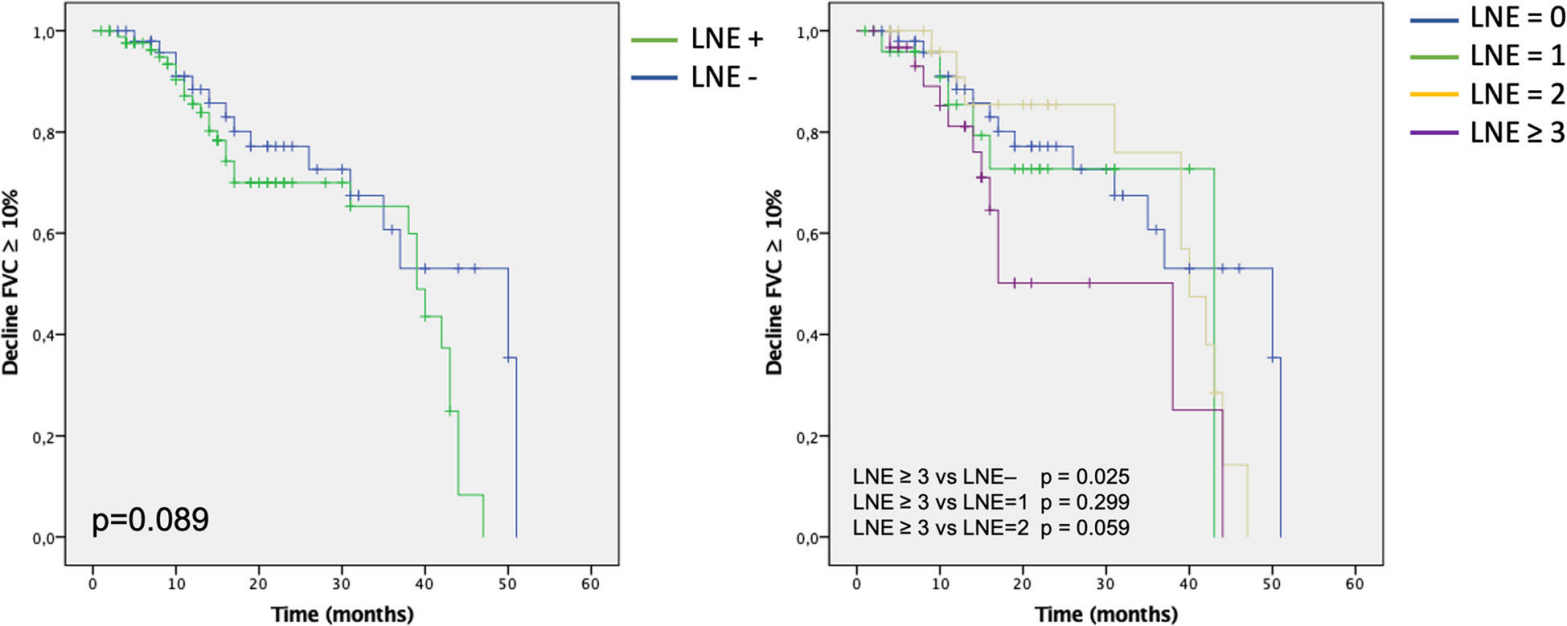
Kaplan-Meyer curves of disease progression, expressed as absolute decline of predicted forced vital capacity (FVC) ≥ 10% for patients with (LNE+) or without (LNE -) mediastinal lymph node enlargement (left) and for patients stratified according to the number of mediastinal enlarged lymph nodes (right). *P* values were obtained via log-rank test

Patients with or without LNE presented similar functional decline as defined as 10% absolute decline in FVC (Fig. 3, Table 2). On the other hand, patients with evidence of 3 or more mediastinal enlarged lymph nodes showed increased disease progression rates both on univariate analysis (HR 2.67, 95% CI 1.19–5.97, *p* = 0.017) and after adjusting for GAP stage (HR 2.99, 95% CI 1.22–7.33, *p* = 0.17) (Table 2).

**Fig. 4:**
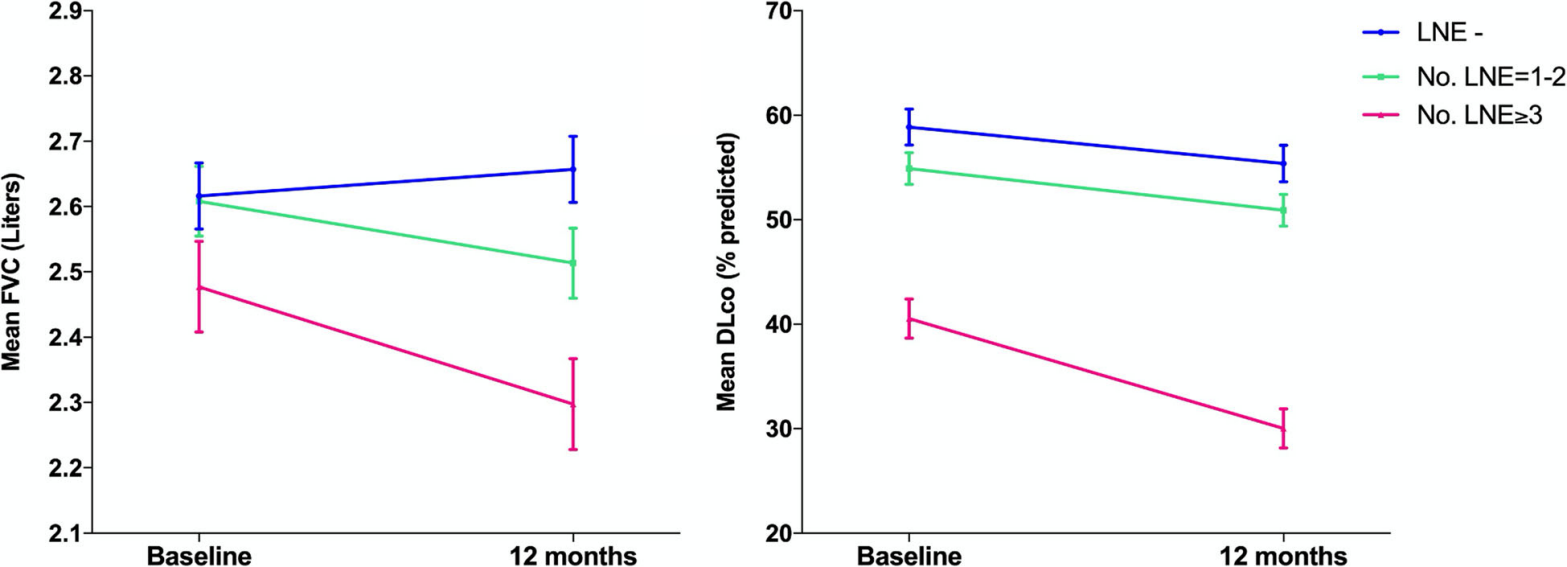
Predicted annual change in Forced Vital Capacity (FVC) and Diffusion Capacity of CO (DLco) for patients with different grade of involvement of mediastinal lymph nodes on baseline CT scan. LNE- = without mediastinal lymph node enlargement; LNE 1–2 = with 1 or 2 enlarged lymph nodes; LNE ≥ 3 = with 3 or more enlarged lymph nodes. Data are predicted mean values with standard error bars. No significant groupXtime interactions were found (*p* = 0.332 and *p* = 0.349 respectively)

In order to further explore the association between mediastinal LNE and change in pulmonary function parameters, the annualized rates of change in absolute FVC and % predicted DLco were compared between patients with different degrees of lymph node involvement at baseline (Fig. 4, Table 3). The rate of decline in FVC increased with the number of mediastinal lymph nodes involved and was largest in patients with 3 or more enlarged lymph nodes (− 178 mL, SE 0.09), especially if compared to patients without LNE, who showed relative stability over 12 months (FVC change − 4 mL, SE 0.07). However, the differences in the rates of change in FVC were not statistically significant as shown by the group-by-time interaction term in the model (*p* = 0.332). The largest decline in DLco occurred in the group with 3 or more enlarged lymph nodes (− 10.5%, SE 2.55) as compared with the other groups, although such difference did not meet statistical significance (group-by-time interaction *p* = 0.349) (Table 3).

**Table 3:** Annualized rates of change in Forced Vital Capacity (liters) and DLco (% predicted) across patients with different grade of involvement of mediastinal lymph nodes on baseline CT scan. LNE- = without mediastinal lymph node enlargement; LNE 1–2 = with 1 or 2 enlarged lymph nodes; LNE ≥ 3 = with 3 or more enlarged lymph nodes. Data are means of predicted values (baseline-12 months) derived from linear mixed models. *p* Values are for group×time interaction. p Values < 0.05 were considered statistically significant

	12-month mean difference (SE)			*p* value
	LNE – *n* = 58	LNE 1–2 *n* = 63	LNE ≥ 3 *n* = 31	
FVC (liters)	−0.04 (0.073)	−0.095 (0.073)	- 0.178 (0.094)	0.332
DLco (% predicted)	−3.49 (2.35)	- 3.99 (2.12)	- 10.5 (2.55)	0.349

### Longitudinal assessment of mediastinal LNE

One hundred seventeen patient cases who had available longitudinal imaging data were assessed for the presence of mediastinal LNE at the follow up CT scan. Average time difference between consecutive CT scans was 17.7 months (SD 10.4). LNE status did not change over time in 101 (86%) of these patients. Among the 48 patients without evidence of mediastinal LNE at baseline, 38 (79%) patients remained without mediastinal LNE at follow up, while among the 69 patients with baseline mediastinal LNE 63 (91%) maintained evidence mediastinal LNE over time. Among the 62 patients with baseline LNE who received antifibrotic treatment, 57 (92%) had mediastinal LNE at follow up. Mediastinal LNE disappeared in only 6 (9%) patients who had LNE at baseline, while 10 (11%) patients who did not have LNE at baseline developed LNE at follow up.

## Discussion

In this study we retrospectively investigated the impact of mediastinal LNE on survival and disease behaviour in patients with IPF followed up at a tertiary ILD centre in Italy. Our findings suggest that a diffuse involvement of mediastinal lymph nodes in IPF patients is an independent predictor of both mortality and functional deterioration in these patients.

Predicting the course of IPF represents a historical challenge for clinicians. Current risk prediction models, robustly developed using data from large randomized clinical trials, may not be suitable for individual prognostication in the real life heterogeneous IPF populations. Furthermore, no parameters have shown to be useful in predicting future trends of functional decline, the hallmark of progressive disease in IPF [13]. Mediastinal lymph node enlargement on CT scan has been recently investigated as a prognostic biomarker in IPF and other fibrotic ILD, for reflecting pathobiological mechanisms of activated immune response that may be relevant to easily stratify patient with at risk of poorer outcome. Sin and collagues [22] demonstrated that mediastinal LNE was a strong, independent predictor of mortality in a retrospective cohort of 132 patients with IPF. Adegunsoye and colleagues [23] nicely reported the association of several features of mediastinal lymphadenopathies with disease severity and higher risk of mortality or hospitalization in patients with a variety of forms of ILD, including IPF, IPAF (interstitial pneumonia with autoimmune features), CTD-ILD (connective tissue disease-associated interstitial lung disease) and unclassifiable ILD, suggesting a role for mediastinal lymph node assessment in ILD prognostication beyond specific etiology.

In our cohort, a “diffuse” lymph node involvement - as defined by 3 or more enlarged mediastinal lymph nodes - was strongly associated with lower survival rates, in line with previous findings. Sin and coworkers [22] demonstrated the impact of mediastinal LNE on survival in an equally-sized IPF cohort with comparable size and similar proportion of patients showing lymph node enlargement on CT. Notably though, the large majority of patients in our study received antifibrotic treatment after diagnosis, suggesting that the presence of multiple mediastinal lymphadenopathies may predict poorer prognosis despite treatment.

Importantly, our study provided first evidence as to the relationships between mediastinal LNE and disease progression expressed by means of functional decline. The involvement of 3 or more lymph nodes involved was found to be associated with an increased risk of disease progression as defined by significant drop (≥10%) in percent predicted FVC, suggesting that diffuse mediastinal LNE represent a marker of more aggressive disease behaviour in these patients. Patients with higher number of lymph nodal stations involved also showed decreased annual trends of pulmonary function and gas diffusion capacity, although the slopes of change were not found significantly different as compared to patients with no or lesser lymph node involvement. Whilst our study was not powered to detect the impact of LNE on continuous change in pulmonary function parameters in a treated IPF population with reduced progression rates, these findings further support the hypothesis that major lymph node involvement predicts future functional deterioration.

The longitudinal evaluation of mediastinal LNE in a subpopulation with follow up imaging data showed that such finding is stable over time in most patients despite currently available antifibrotic treatment, which seems to preclude the role of LNE as a marker of response to treatment. Indeed, further studies are needed to clarify the pathobiological mechanisms at the basis of this prevalent finding in IPF patients. The study of histological features of LNE may help shed a light on the immune processes involved and whether a subset of patients exists where such pathways are paramount for driving disease progression.

The main limitations of our study are represented by its retrospective nature and the small size of our cohort. Nevertheless, the study was adequately powered based on previously reports of mortality hazard ratio for IPF patients with LNE [22], which increases the robustness of our results. Another theoretical limitation consists in the heterogeneity of CT reconstruction protocols and contrast medium administration, as the exams have been performed in different centres.

## Conclusions

We demonstrated that the presence of diffuse mediastinal lymphadenopathy on CT scan is independently associated with higher risks of mortality and clinically significant disease progression in a cohort of IPF patients despite antifibrotic treatment. Altogether, our results support the utility of mediastinal LNE in the risk stratification of the heterogeneous IPF population. Ongoing trials and future research will eventually clarify the pathobiology of mediastinal lymphadenopathy in IPF and if such finding may be useful to identify patients responding to treatments targeting specific immune pathways.

## Data Availability

The datasets used and/or analysed during the current study are available from the corresponding author on reasonable request.

## Abbreviations

LNE: Lymph Node Enlargement
IPF: Idiopathic Pulmonary Fibrosis
FVC: Forced Vital Capacity
HRCT: High-resolution computed tomography
ILD: Interstitial Lung Disease
PFTs: Pulmonary Function Tests
DLco: Diffusion capacity for carbon monoxide
GAP: Gender/Age/Physiology
IPAF: Interstitial pneumonia with autoimmune features
CTD-ILD: Connective tissue disease-associated interstitial lung disease
